# *GAT1* Gene, the GATA Transcription Activator, Regulates the Production of Higher Alcohol during Wheat Beer Fermentation by *Saccharomyces cerevisiae*

**DOI:** 10.3390/bioengineering8050061

**Published:** 2021-05-08

**Authors:** Ya-Ping Wang, Lin Liu, Xue-Shan Wang, Kun-Qiang Hong, Li-Hua Zhang, Zhong-Guan Sun, Dong-Guang Xiao

**Affiliations:** 1 Key Laboratory of Industrial Fermentation Microbiology, Ministry of Education, Tianjin Industrial Microbiology Key Laboratory, College of Biotechnology, Tianjin University of Science and Technology, Tianjin 300457, China; wangyapingyeah@163.com; 2 College of Life Science, Zaozhuang University, Zaozhuang 277160, China; zzxyliulin@163.com; 3 College of Food Science and Pharmaceutical Engineering, Zaozhuang University, Zaozhuang 277160, China; wangxueshan_1987@163.com (X.-S.W.); chinazhanglh@163.com (L.-H.Z.); 4 Department of Biochemical Engineering, School of Chemical Engineering and Technology, Tianjin University, Tianjin 300072, China; leo_hong92@163.com; 5 Key Laboratory of Wuliangye-Favor Liquor Solid-State Fermentation, China National Light Industry, Yibin 644000, China

**Keywords:** *Saccharomyces cerevisiae*, higher alcohol, nitrogen catabolite repression, GATA factors, *GAT1*

## Abstract

Uncoordinated carbon-nitrogen ratio in raw materials will lead to excessive contents of higher alcohols in alcoholic beverages. The effect of *GAT1* gene, the GATA transcription activator, on higher alcohol biosynthesis was investigated to clarify the mechanism of *Saccharomyces cerevisiae* regulating higher alcohol metabolism under high concentrations of free amino nitrogen (FAN). The availability of FAN by strain SDT1K with a *GAT1* double-copy deletion was 28.31% lower than that of parent strain S17, and the yield of higher alcohols was 33.91% lower. The transcript levels of the downstream target genes of *GAT1* and higher alcohol production in the double-copy deletion mutant suggested that a part of the effect of *GAT1* deletion on higher alcohol production was the downregulation of *GAP1*, *ARO9*, and *ARO10*. This study shows that GATA factors can effectively regulate the metabolism of higher alcohols in *S. cerevisiae* and provides valuable insights into higher alcohol biosynthesis, showing great significance for the wheat beer industry.

## 1. Introduction

Wheat beer must be top-fermented, produced from at least 50% malted wheat, occasionally added with non-germinated malt, and has an original gravity of at least 11 °P [1]. Alcoholic beverages usually have an exquisite pure-white beer head, mellow and smooth taste, and may taste slightly bitter [2,3]. Owing to this unique aroma, wheat beer is beloved by consumers, particularly in Bavaria, Belgium, and Austria [4].

A major characteristic of the flavor maturation of beer is the well-adjusted content of volatile compounds. Higher alcohols and related esters together contribute to the overall flavor of wheat beer. An appropriate content and proportion of higher alcohols gives beer consistency and a full-bodied taste. Given its protein-rich raw materials and high fermentation temperature, the total content of higher alcohols in wheat beer usually exceeds 300 mg/L. Excessive amounts of higher alcohols result in an unpleasant flavor of wheat beer and can cause headache and intoxication in consumers.

During beer fermentation, higher alcohols are mainly generated by *S. cerevisiae*. These compounds are synthesized via de novo synthesis from carbon sources and the degradation of branched-chain amino acids. In 1904, the biochemist Ehrlich discovered that branched amino acids can be catabolized to produce higher alcohols in yeast [5]. The metabolism of higher alcohols has been studied for more than a century. However, only genes directly involved in higher alcohols metabolism pathways have received considerable attention [6,7,8,9,10,11]. The influence of regulatory genes, such as those controlling the carbon and nitrogen metabolism, on higher alcohols synthesis was all but ignored [12,13].

In yeast, the transcription of genes that mainly encode non-preferred nitrogen source metabolic enzymes is regulated by GATA factor, which includes *GAT1* and *GLN3* (two activators), and *DAL80* and *GZF3* (two inhibitors) [14]. In the presence of preferred nitrogen sources, such as glutamine, asparagine, or ammonium, Gln3p and Gat1p were isolated in cytoplasm and cannot participate in the transcriptional regulation of target genes [15]. Lee and Hahn observed that the binding of Gat1p with *GAP1*, *ARO9*, *ARO10,* and *ARO80* promoters was significantly enhanced under the condition of poor nitrogen sources [15]. *GAP1* mediates the active transport of all-natural amino acids and numerous amino acid analogs into the cells [16]. *ARO9* catalyzes the transamination of tryptophan, phenylalanine, and tyrosine during their catabolism. *ARO10*, a decarboxylase, is induced by tryptophan in the same way as *ARO9* [17]. In the presence of aromatic amino acids, *ARO80* activates the expression of *ARO9* and *ARO10* genes [15]. However, the mechanism of *GAT1* regulating the metabolism of several higher alcohols has not been investigated. The mechanism by which *GAT1* regulates the metabolism of higher alcohols during wheat beer fermentation also remains unclear.

In this study, mutants of top-fermenting yeast with *GAT1* deletion were constructed. Their effects on higher alcohol metabolism and other fermentation performance parameters in wheat beer production were investigated. Moreover, engineered strains with double-copy deletions of genes targeted by *GAT1* were constructed, and the effects of these mutants on higher alcohol content in wheat beer were determined.

## 2. Materials and Methods

### 2.1. Strains and Plasmids

Table 1 lists all the strains and plasmids used in the current study.

### 2.2. Medium and Culture Conditions

Seed medium: Luria–Bertani broth was used for the culture of *E. coli* [20]. Yeast extract peptone dextrose (YEPD) medium (2% glucose, 2% peptone, and 1% yeast extract) was used for the culture of yeast strains. The YEPD medium with 40 mg/L G418 antibiotic was used to screen the transformed recombinant strains with the integrated *KanMX* gene. The YEPD medium with 500 mg/L Zeocin was used to screen the transformed recombinant strains with *KanMX* gene, which was successfully eliminated by selection. The G418 antibiotic and Zeocin were purchased from Promega (Madison, WI, USA). All solid media were added with 2% agar (Solarbio, Beijing, China).

Fermentation medium: For the fermentation medium using wort, the 12 °P wort was prepared as described in a previous report [21].

Seed culture: Yeast strains were cultured in triangle flasks with 50 mL wort (12 °P), sealed with eight layers of gauze, and incubated for 36 h at 24 °C statically. After the enrichment culture, the yeast liquid was centrifuged (4226× *g*, 3 min) and transferred to a fermentation bottle to obtain 0.5% yeast pulp.

Fermentation condition: The fermentation experiment was implemented in a 250 mL flask with 150 mL 12 °P wort. The flasks were sealed by rubber stoppers with a CO_2_ outlet. The second precultured yeast seed liquid was inoculated in the fermentation medium. The inoculum density was about 6 × 10^7^ cells/mL.

### 2.3. Yeast Transformation and Screening

Gene knockout was realized by integrating the *KanMX* cassette to substitute for the target gene with S17 as the parental strain. Table S1 lists the primers used in this study, which were designed on the basis of the *S. cerevisiae S288c* genome sequence in the National Center for Biotechnology Information website (http://www.ncbi.nlm.nih.gov/, accessed on 15 June 2019). Upstream/downstream homologous fragments of target genes were amplified by polymerase chain reaction (PCR) using U-F/U-R and D-F/D-R as primers and S17 genome as the template. The *KanMX* gene segment was amplified by PCR using Kan-F/Kan-R as primers and pUG6 plasmid as the template. The homologous recombination of the fragments and yeast genome was implemented using the LiAc/SS carrier DNA/PEG method [22]. YEPD solid medium mixed with G418 was used to preliminarily screen the transformants. The exact integration of the *KanMX* cassette in a single colony was verified by diagnostic PCR with verification primers (1-F/1-KanR and 2-KanF/2-R). Using the Cre/loxP reporter rescue system, the PSH-Zeocin plasmid was transformed into the recombinant strains by LiAc/SS carrier DNA/PEG method, and the primer K-U/K-D was used to screen and obtain the transformants for *KanMX* resistance marker elimination through PCR verification. Then, the obtained transformants were subcultured to discard the free plasmid of PSH-Zeocin, and the primers Zn-U/Zn-D were used to screen the recombinant strains, successfully discarding the plasmid of PSH-Zeocin through PCR verification. Finally, the required *S. cerevisiae* mutant was obtained.

### 2.4. Quantitative Real-Time PCR (RT-qPCR)

Yeast strains were cultured at 20 °C in the fermentation medium for 36 h. Total RNA was extracted by yeast RNA isolation kit and reverse transcribed with PrimeScript™ RT reagent kit. RT-qPCR was used to detect the change in gene expression levels with SYBR^®^Premix Ex TaqTM II test kit (Tli RNaseH Plus). The kits were purchased from Takara Biotechnol (Dalian, China). The PCR procedure was set as reported by Li et al. [23]. The primers (gene-F/gene-R) synthesized by GENEWIZ (Suzhou, China) are listed in the Supplementary Material. The results were analyzed quantitatively using 2^−ΔΔCt^ method, and *UBC6* was used as the housekeeping gene.

### 2.5. Fermentation Trials

After the fermentation had started, the wort was weighed every 12 h. When the weight loss was less than 0.1 g, fermentation was regarded as complete. After the fermentation was completed, the ethanol content and real fermentation degree were determined by the density bottle method. Residual sugar was measured by a handheld digital hydrometer (LH-B55; LoHand Biological, Hangzhou, China). Free amino nitrogen (FAN) concentration was determined in accordance with the ninhydrin-based standard method, which was demonstrated by the European Brewery Convention (EBC Analytica, 1998). The concentrations of higher alcohols and other flavor substances in the distillate were determined by gas chromatography (GC) in accordance with Ma’s report [20].

### 2.6. Statistical Analysis

The experiments were repeated thricely. The experimental data were represented as mean ± the standard error of the mean. The significance of the difference between experimental and control groups was analyzed using *t*-test (^★★^ *p* < 0.01, ^★^ *p* < 0.05).

## 3. Results

### 3.1. Construction of GAT1 Deleted Strains

**Figure 1:**
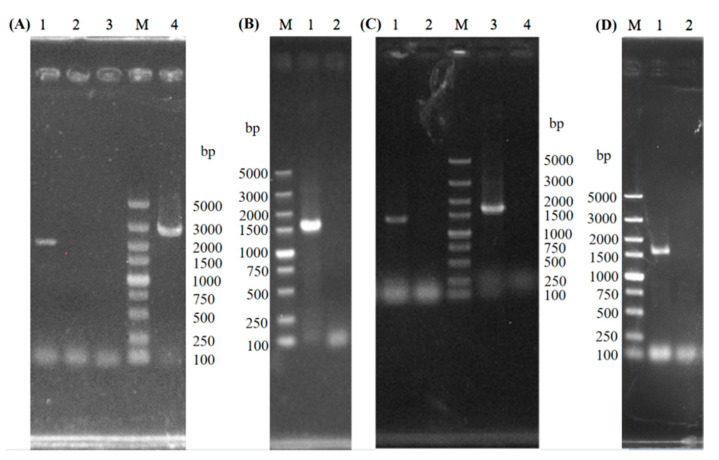
PCR verification of the recombinants. M: DL5000 DNA Marker; (**A**) lanes 1-2: PCR amplification results from SST1 and S-17 genome using the primers SST1-1-F and SST1-1-KanR; lanes 3–4: PCR amplification results from S-17 and SST1 genome using the primers SST1-2-KanF and SST1-2-R. (**B**) lanes 1–2: PCR amplification results from SST1 and SST1K genome using the primers K-U and K-D. (**C**) lanes 1-2: PCR amplification results from SDT1 and SST1K genome using the primers SDT1-1-F and SDT1-1-KanR; lanes 3–4: PCR amplification results from SDT1 and SST1K genome using the primers SDT1-2-KanF and SDT1-2-R. (**D**) lanes 1–2: PCR amplification results from SDT1 and SDT1K genome using the primers K-U and K-D.

The *GAT1* allele on one chromosome of S17 was replaced with the fragment GAT1A, GAT1B, and the *KanMX* cassette *loxP-KanMX-loxP* to construct the single-copy knockout strain SST1 through homologous recombination (Figure 1A). PCR was used to verify the mutant strain SST1, and 2225- and 2887 bp bands were obtained (lanes 1 and 4 in Figure 1A, respectively) for SST1. These results indicate that the recombinant strain SST1 with a single-copy deletion of *GAT1* was successfully constructed. Cre recombinase was expressed to excise the *KanMX* cassette with the plasmid pSH-Zeocin introduced to obtain the recombinant strain SST1K. The results of PCR validation for the recombinant strain SST1K (lane 2 in Figure 1B) indicated that *KanMX* in SST1 was successfully removed.

The fragments GAT1A2, *loxP-KanMX-loxP*, and GAT1B2 were introduced into SST1K to obtain the recombinant strain SDT1 with a couple-copy deletion of *GAT1*. By using the same method as for SST1K, *KanMX* gene was removed from SDT1, and the recombinant strain SDT1K was constructed. Amplified fragments of 1349 and 1632 bp were obtained for SDT1 (lanes 1 and 3 in Figure 1C, respectively), validating the successful construction of SDT1. No PCR amplified fragments were found in lane 2 (Figure 1D), which indicates the successful removal of *KanMX* in SDT1.

### 3.2. Effects of GAT1 Deletion on Higher Alcohol Metabolism

**Figure 2:**
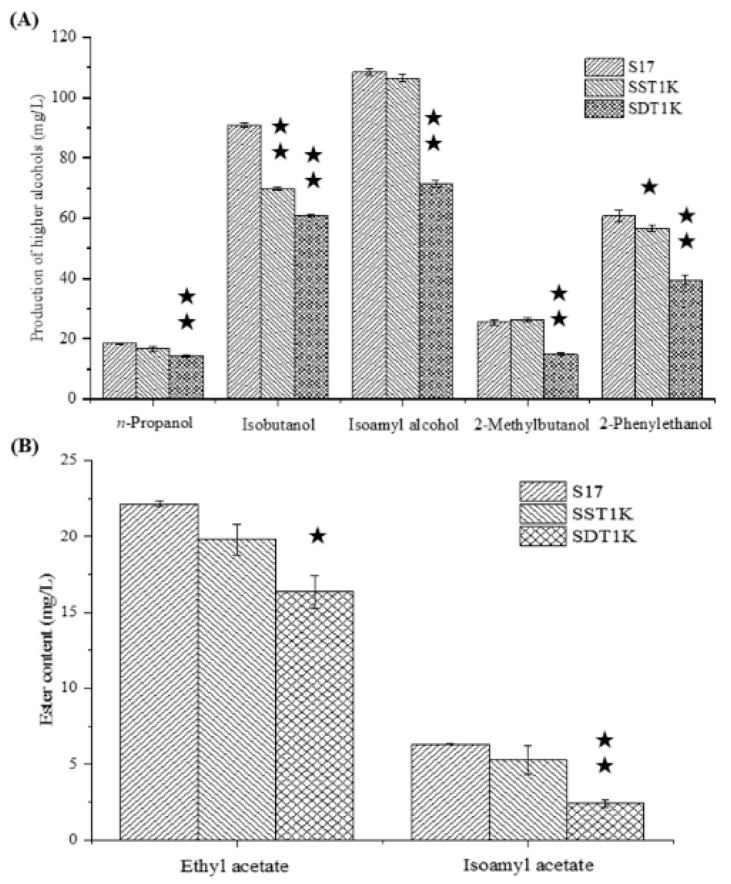
GC analysis results of the samples. (**A**) Higher alcohol production by *GAT1* deletion mutants and parental strain S17. (**B**) Esters production by *GAT1* deletion mutants and parental strain S17. Data are the averages of three independent experiments. Error bars represent ± SD. ^★★^ *p* < 0.01, ^★^ *p* < 0.05.

The effects of the single- and couple-copy deletions of *GAT1* on higher alcohol metabolism were investigated in all-wheat beer fermentation. Figure 2 presents the GC analysis results of the samples. The single-copy deletion of *GAT1* decreased the contents of isobutanol and 2-phenylethanol significantly. The isobutanol and 2-phenylethanol produced by SST1K reached 69.86 and 56.64 mg/L, respectively. These values represented 23.13% (isobutanol) and 6.75% (2-phenylethanol) reductions compared with those of strain S17 (*p* < 0.05). The double-copy deletion of *GAT1* decreased the higher alcohol content to a high degree and the content of ethyl acetate and isoamyl acetate significantly. The higher alcohol content of SDT1K was 201.05 mg/L, representing 33.91% reduction compared with the original strain S17. The concentration of 2-methylbutanol decreased the most with a value of up to 41.89%, followed by 2-phenyl ethanol, isoamyl alcohol, isobutanol, and *n*-propanol, with values of 34.96%, 34.07%, 33.17%, and 22.04%, respectively. The expression level of *GAT1* gene decreased, the ability of yeast cells to regulate nitrogen metabolism was weak, and yeast could not effectively utilize non-preferred nitrogen sources, such as branched-chain and aromatic amino acids, in the culture medium, which led to a significant decrease in the higher alcohol synthesis ability of yeast, especially for isobutanol, isoamyl alcohol, and 2-phenyl ethanol.

*GAT1* and *GLN3* increase the expression levels of nitrogen catabolite repression (NCR)-sensitive genes under nitrogen-limiting conditions to enhance the utilization of branched chains and aromatic amino acids [14]. The utilization of FAN was inhibited by *GAT1* inactivation (Table 2), and the content of higher alcohols was significantly reduced (Figure 2). Effective restriction of *GAT1* activity will reduce the utilization of nitrogen sources by *S. cerevisiae*, thus affecting the synthesis of higher alcohols. In conclusion, *GAT1* plays an important role in regulating the expression activity of NCR-sensitive genes and the utilization of FAN by *S. cerevisiae*.

### 3.3. Effects of GAT1 Deletion on the Growth and Fermentation Performance of Yeast

**Figure 3:**
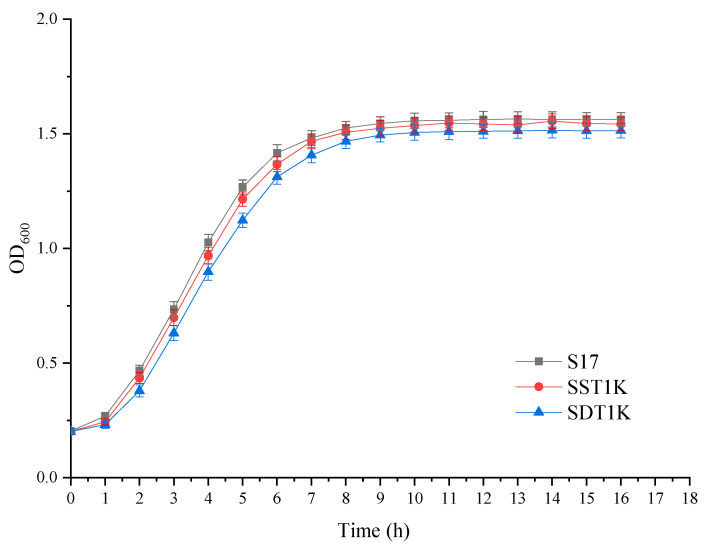
Growth curves of *GAT1* deletion mutants and the parental strain S17. Growth curves were plotted using a Bioscreen Automated Growth Curves analysis system. The yeast cells were cultured at 30 °C in YEPD medium, and the optical density (OD_600_) of the fermentation broth was detected at different times. Data are the average of three independent experiments. Values are means and standard errors.

The growth and fermentation performance were investigated to assess the effects of *GAT1* deleted on top-fermenting yeast. Compared with S17, the growth rate and final cell density of SST1K and SDT1K slightly differed (Figure 3). As shown in Table 2, the knockout strains showed similar fermentative capabilities to their parental strains except in the consumption of FAN. The contents of FAN consumed by SST1K and SDT1K were 123.45 and 100.08 mg/L, respectively, which were both less than that consumed by S17 (140.24 mg/L). The results showed that *GAT1* deletion had no significant effect on the growth and fermentation performance of S17. In the case of sufficient preferential nitrogen sources, such as ammonium, glutamine, and asparagine, *GAT1* is not an essential gene for the growth and metabolism of *S. cerevisiae*, but it plays an active role in the anabolism of higher alcohols.

### 3.4. mRNA Levels of Genes Related to Higher Alcohol Metabolism

**Figure 4:**
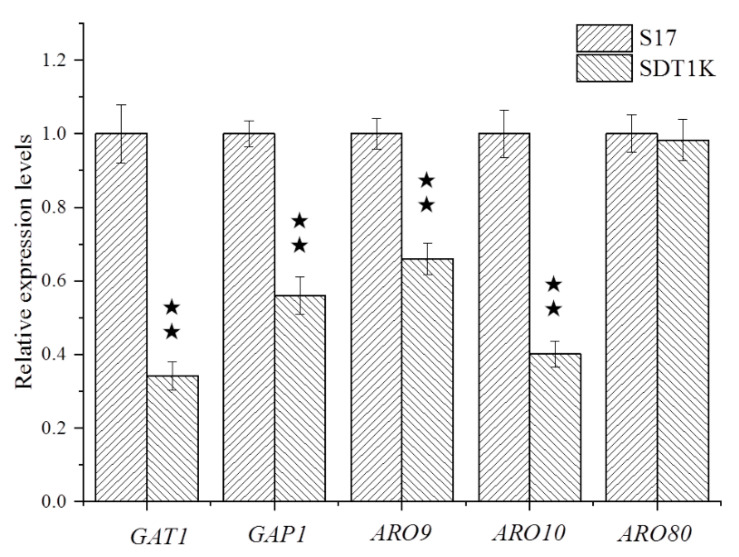
Determination of gene transcription levels in parental strain S17 and the mutant strain SDT1K. Total RNA of parental strains and mutants were isolated and used as the template to obtain the cDNA of *GAT1*, *GAP1*, *ARO9*, *ARO10* and *ARO80*, which was used to determine the relative expression level of the corresponding gene by RT-qPCR. Data are the average of three independent experiments. Error bars represent ± SD. ^★★^ *p* < 0.01.

Given that the contents of higher alcohols all decreased significantly in SDT1K, the expression levels of the *GAT1* gene and its target genes related to the Ehrlich pathway (*GAP1*, *ARO9*, *ARO10*, and *ARO80*) in the recombinant strain SDT1K were quantified. The results of RT-PCR (Figure 4) showed that the expression levels of *GAT1* in SDT1K (with two copies of *GAT1* deleted) decreased by 66% compared with that of strain S17 (*p* < 0.05). The deletion of *GAT1* gene effectively reduced the transcription level of this gene in *S. cerevisiae* S17, thereby reducing the content of Gat1p and affecting its metabolic activity. Therefore, the deletion of *GAT1* gene can effectively reduce the nitrogen metabolism and higher alcohol synthesis ability of S17. Moreover, the *GAT1* gene deletion resulted in 44%, 36%, and 59% decreases in the expression levels of *GAP1*, *ARO9*, and *ARO10*, respectively. However, no significant change was observed in the expression level of *ARO80*. *GAT1* gene can affect the nitrogen metabolism and higher alcohol synthesis ability of strain S17 by regulating the transcription levels of *GAP1*, *ARO9*, and *ARO10* genes. This finding may be due to the amount of gene expression changes during fermentation. After fermentation for 36 h, the time for expression of *ARO80* had passed. Lee and Hahn observed that in the case of insufficient nitrogen sources, the expression level of *GAP1*, *ARO9*, *ARO10*, and *ARO80* in *S. cerevisiae* significantly reduced after deleting the *GAT1* gene of BY4741 [15]. These results demonstrated that the regulation of NCR-sensitive genes by *GAT1* differs based on the strain and fermentation conditions in *S. cerevisiae*. The authentic relationship between *GAT1* and NCR-sensitive genes at the transcriptional level needs to be clarified in more detail.

### 3.5. Effects of Deleting Target Genes of GAT1 on the Content of Higher Alcohols in Wheat Beer

**Figure 5:**
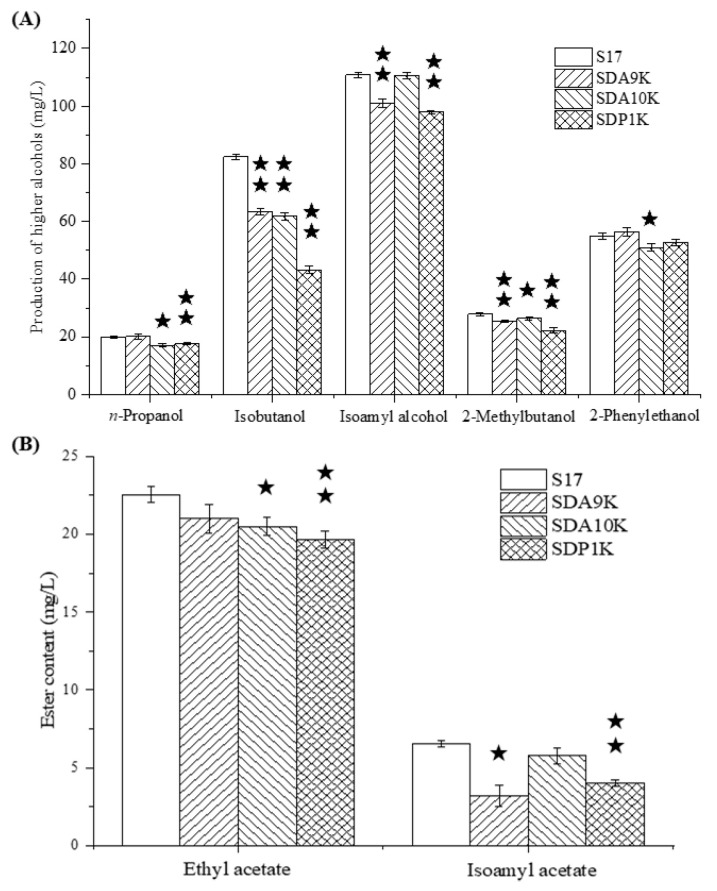
(**A**) Higher alcohol production by *GAT1* target genes double allele deletion mutants and parental strain S17. (**B**) Esters production by *GAT1* target genes double allele deletion mutants and parental strain S17. Data are the averages of three independent experiments. Error bars represent ± SD. ^★★^ *p* < 0.01, ^★^ *p* < 0.05.

The recombinant strains SDP1K, SDA9K, and SDA10K have double-copy deletions of *GAP1*, *ARO9*, and *ARO10*, which are similar to the recombinant strain SDT1K. The effects of modifications on the formation of higher alcohols were investigated in all-wheat beer fermentation. The effects of *GAP1*, *ARO9*, and *ARO10* double-copy deletions on the formation of higher alcohols differed (Figure 5A).

Compared with the parental strain, the *GAP1* double-copy deletion mutant SDP1K yielded 11.73% less *n*-propanol, 47.57% less isobutanol, 11.70% less isoamyl alcohol, 20.09% less 2-methylbutanol, and 3.85% less 2-phenylethanol. These results indicated that Gap1p inactivation significantly reduced the synthesis ability of top-fermenting yeast for higher alcohols, especially isobutanol. Our preliminary study revealed that the single deletion of *GAP1* also affected the higher alcohol synthesis ability and α-amino nitrogen utilization ability of *S. cerevisiae* [21]. Chiva et al. indicated that *GAP1* permease not only acts as an amino acid transporter but also as an amino acid sensor in *S. cerevisiae* [24].

Similar to the recombinant strain SDP1K, the *ARO10* double-copy deletion strain SDA10K exhibited 14.20% decrease in *n*-propanol, 24.99% decrease in isobutanol, 0.31% decrease in isoamyl alcohol, 5.23% decrease in 2-methylbutanol, and 7.27% decrease in 2-phenylethanol production compared with the original strain S17. *ARO10* gene encodes an efficient phenylpyruvate decarboxylase that possibly plays a prominent role in aromatic amino acid catabolism [25]. Dickinson et al. considered that Aro10p was the minor decarboxylase responsible for the 6% flux in leucine degradation, with leucine as the only nitrogen source; Aro10p sufficiently catalyzes the catabolism of isoleucine to 2-methylbutanol with isoleucine as the only nitrogen source [26,27]. However, at high concentrations of FAN, the production of n-propanol and isobutanol in *S. cerevisiae* S17 decreased significantly with *ARO10* gene double deletion, which may be caused by the decrease in Aro10p catalysis that led to the competitive utilization of decarboxylase. Furthermore, the 2-phenylethanol content of SDA10K decreased significantly compared with that of S17. This finding was consistent with the result reported by Yin et al., who observed higher expression levels of *ARO10* in *S. cerevisiae*, which exhibited high 2-phenylethanol production during fermentation, than in yeast strains with low 2-phenylethanol production rates [28].

Different from the recombinant strains SDP1K and SDA10K, the *ARO9* double-copy deletion strain SDA9K exhibited a 1.02% increase in *n*-propanol, 23.22% decrease in isobutanol, 8.95% decrease in isoamyl alcohol, 9.23% decrease in 2-methylbutanol, and 2.69% increase in 2-phenylethanol production. By contrast, Kim et al. showed that the overexpression of *ARO9* in *S. cerevisiae* W303 can increase the yield of 2-phenethyl alcohol. *ARO9* gene encodes aromatic aminotransferase II, which catalyzes the transamination of phenylalanine and is induced by phenylalanine as an amino donor [29,30]. One possible explanation for this result is that the inactivation of Aro9p may lead to an increase in the expression of its homologue Aro8p (aromatic aminotransferase I), which will promote the synthesis of 2-phenethyl alcohol [28,31]. Notably, the isobutanol synthesis capacity of recombinant strain SDA9K decreased significantly, and this finding may be related to the reduced production of keto-isovalerate. However, further scientific evidence is needed to prove this assumption.

These results indicate that the *GAP1* gene has a greater influence on the production of higher alcohols than *ARO9* or *ARO10*. Interestingly, double-copy deletion of any of the three genes decreased the production of isobutanol and 2-methylbutanol. A double-copy deletion of *GAP1* resulted in a decrease in the isobutanol and 2-methylbutanol synthesis abilities of the top-fermenting yeast strain, which may be due to the decrease in the valine and isoleucine utilization ability. However, this idea needs to be confirmed in more detailed studies. Dickinson et al. proved that Aro10p was sufficient to catalyze the catabolism of isoleucine to 2-methylbutanol with isoleucine as the only nitrogen source [26]. Aro9p directly converts phenylalanine into 2-phenythyl alcohol through the Ehrlich pathway [16]. However, the relationship between Aro9p and isobutanol or 2-methylbutanol metabolism regulation has not been reported, which may also be related to the type of nitrogen source in the culture system. The effects of mass concentrations of nitrogen, nitrogen sources, and C/N mass ratio on higher alcohol synthesis in *S. cerevisiae* need to be reconsidered.

The ethyl acetate and isoamyl acetate in all-wheat beer were also quantified (Figure 5B). The ethyl acetate and isoamyl acetate contents of SDP1K were 19.06 and 4.01 mg/L, respectively. The values represent reductions of 15.52% (ethyl acetate) and 38.76% (isoamyl acetate). The contents in SDA10K decreased by 9.12% (ethyl acetate) and 11.91% (isoamyl acetate). The ethyl acetate and isoamyl acetate contents of SDA9K decreased by 6.93% and 32.55%, respectively, compared with those of strain S17. These results showed that ester synthesis by the recombinant strains showed the same trend as higher alcohol synthesis. The recombinant strain SDP1K had the most significant effect on ethyl acetate synthesis, followed by SDA10K and SDA9K. Thus, when the yield of isoamyl alcohol decreases, the synthesis of isoamyl acetate also reduces.

## 4. Discussion

Given the high content of fermentable sugar and FAN in the fermentation materials of alcoholic beverages, the problem of excessive higher alcohols in alcoholic beverages is widespread. *S. cerevisiae* synthesizes higher alcohols mainly through amino acid catabolism (Ehrlich pathway) and pyruvate anabolism pathways; the expression levels of genes in these metabolic pathways have a decisive effect on higher alcohol synthesis [32,33,34]. When the contents of fermentable sugar and FAN in the culture system are unusually sufficient, all higher alcohol anabolic pathways are in an active state consistently [23,35,36]. Genetic modification of genes in the higher alcohol metabolism pathway may not yield the desired results, and the metabolic regulation genes of carbon and nitrogen sources should be considered. GATA factors, two activators (*GAT1* and *GLN3*), and two repressors (*DAL80* and *GZF3*) can regulate NCR-sensitive transcription presumably via their competitive binding to the GATA sequences [14]. In this study, when both copies of the *GAT1* gene were inactivated, the utilization of FAN by *S. cerevisiae* decreased significantly, whereas the other fermentation properties showed no significant change. The synthesis ability of recombinant strain SDT1K for higher alcohols significantly decreased. The ability to synthesize acetate was also reduced markedly. These results showed that genetic modification of *GAT1* gene, a regulator of nitrogen metabolism, can regulate higher alcohol metabolism, whereas other GATA factors may play an equally important role. Lee and Hahn also observed that *GAP1*, *ARO9*, *ARO10*, and *ARO80* expression levels were significantly reduced after deleting *GLN3* and *GAT1* genes encoding GATA transcription activators [15]. The mRNA levels of the target genes of *GAT1*, which are related to the Ehrlich pathway, were measured, and the corresponding recombinant strains with double-copy deletions were constructed to reveal the molecular mechanism of *GAT1* gene regulating higher alcohol metabolism in *S. cerevisiae*. The results suggested that a part of the effect of *GAT1* deletion on higher alcohol production was attributed to the downregulation of *GAP1*, *ARO9*, and *ARO10*. *ARO80*, a regulatory gene of *ARO9* and *ARO10*, was ignored, possibly because this regulation mode enables *S. cerevisiae* to regulate the metabolism of higher alcohols directly and efficiently.

## 5. Conclusions

At high concentrations of FAN, genetic modification of *GAT1*, a regulator of nitrogen metabolism, can alter the ability of *S. cerevisiae* to produce higher alcohols by regulating the transcriptional levels of *GAP1, ARO9*, and *ARO10*. Interestingly, the *ARO80* gene did not participate in the metabolic regulation of higher alcohols. Our work reports the relationship between *GAT1* and the content of higher alcohols in wheat beer. We clarified the mechanism by which *GAT1* regulates the metabolism of higher alcohols in *S. cerevisiae* at high concentrations of FAN. Almost all the fermentation processes of alcoholic beverages were carried out under the condition of extremely high concentration of carbon and/or the preferred nitrogen sources. This study provides guiding significance to reveal the regulation mode of higher alcohol metabolism in *S. cerevisiae* under the condition of high-concentration nutrients to control the higher alcohol contents in beverages.

## Figures and Tables

**Table 1:** Strains and plasmids used in the current study.

Strains or Plasmids	Relevant Characteristic	References or Source
Strains		
S17, CICC1929	Wild-type top-fermenting industrial brewer’s strain	This study
*E. coli* DH5α	Host of plasmid	Stratagene
Transformants		
SST1	S-17*GAT1(n-1)/*Δ*GAT1::loxP-KanMX-loxP*	This study
SST1K	S-17*GAT1(n-1)/*Δ*GAT1::loxP::loxP*	This study
SDT1	S-17*GAT1(n-2)/*Δ*GAT1::loxP-KanMX-loxP*	This study
SDT1K	S-17*GAT1(n-2)/*Δ*GAT1::loxP::loxP*	This study
SSP1	S-17*GAP1(n-1)/*Δ*GAP1::loxP-KanMX-loxP*	This study
SSP1K	S-17*GAP1(n-1)/*Δ*GAP1::loxP::loxP*	This study
SDP1	S-17*GAP1(n-2)/*Δ*GAP1::loxP-KanMX-loxP*	This study
SDP1K	S-17*GAP1(n-2)/*Δ*GAP1::loxP::loxP*	This study
SSA9	S-17*ARO9(n-1)/*Δ*ARO9::loxP-KanMX-loxP*	This study
SSA9K	S-17*ARO9(n-1)/*Δ*ARO9::loxP::loxP*	This study
SDA9	S-17*ARO9(n-2)/*Δ*ARO9::loxP-KanMX-loxP*	This study
SDA9K	S-17*ARO9(n-2)/*Δ*ARO9::loxP::loxP*	This study
SSA10	S-17*ARO10(n-1)/*Δ*ARO10:: loxP-KanMX-loxP*	This study
SSA10K	S-17*ARO10(n-1)/*Δ*ARO10::loxP::loxP*	This study
SDA10	S-17*ARO10(n-2)/*Δ*ARO10::loxP-KanMX-loxP*	This study
SDA10K	S-17*ARO10(n-2)/*Δ*ARO10::loxP::loxP*	This study
**Plasmids**		
pUG6	Kan ^r^, containing *loxP-KanMX-loxP* disruption cassette	[18]
pSH-Zeocin	Zeo ^r^, Cre recombinant enzyme expression vector	[19]

**Table 2:** Fermentation performances of the original strain S17 and its recombinants.

Strains	Weight Loss of CO_2_ (g)	Ethanol (% *v*/*v*, 20 °C)	Residual Sugar (g/L)	Residual FAN (mg/L)	Real Fermentation Degree (%)
S17	5.77 ± 0.058	4.04 ± 0.0208	5.93 ± 0.058	141.88 ± 0.235	68.37 ± 0.112
SST1K	5.70 ± 0.000	4.03 ± 0.0451	6.03 ± 0.058	158.67 ± 0.359 ^★★^	68.25 ± 0.190
SDT1K	5.77 ± 0.058	4.04 ± 0.0513	5.90 ± 0.000	182.04 ± 0.136 ^★★^	68.22 ± 0.378

Data are the average of three independent experiments ± the standard deviation. Significant difference of *GAT1* deletion strains (SST1K and SDT1K) from the parental strain was confirmed by Student’s *t*-test. ^★★^ *p* <0.01.

## Data Availability

The data used to support the findings of the current study are available from the corresponding author on reasonable request.

## Supplementary Materials

The following are available online at https://www.mdpi.com/article/10.3390/bioengineering8050061/s1, Table S1: Oligonucleotides used in this study for the construction of gene deletions.
