# Supplementary material for: GAT1 Gene, the GATA Transcription Activator, Regulates the Production of Higher Alcohol during Wheat Beer Fermentation by Saccharomyces cerevisiae
Source: Bioengineering (Basel). 2021 May 8;8(5):61. doi: 10.3390/bioengineering8050061 (PMC8151594; doi:10.3390/bioengineering8050061)
Supplement: Supplementary file 1 [file bioengineering-08-00061-s001.zip › bioengineering-1141674-supplementary.pdf]

bioengineering

MDPI

Supplementary materials

GAT1 Gene, the GATA Transcription Activator, Regulates the Production of Higher Alcohol during Wheat Beer Fermentation by *Saccharomyces cerevisiae*

Ya-Ping Wang [1,†], Lin Liu [2,†], Xue-Shan Wang [3], Kun-Qiang Hong [4], Li-Hua Zhang [3], Zhong-Guan Sun [3,1,5,*] and Dong-Guang Xiao [1,*]

1 Key Laboratory of Industrial Fermentation Microbiology, Ministry of Education, Tianjin Industrial Microbiology Key Laboratory, College of Biotechnology, Tianjin University of Science and Technology, Tianjin 300457, China; wangyapingyeah@163.com
2 College of Life Science, Zaozhuang University, Zaozhuang 277160, China; zzxyliulin@163.com
3 College of Food Science and Pharmaceutical Engineering, Zaozhuang University, Zaozhuang 277160, China; wangxueshan_1987@163.com (X.-S.W.); chinazhanglh@163.com (L.-H.Z.)
4 Department of Biochemical Engineering, School of Chemical Engineering and Technology, Tianjin University, Tianjin 300072, China; leo_hong92@163.com
5 Key Laboratory of Wuliangye-favor Liquor Solid-State Fermentation, China National Light Industry, Yibin 644000, China
* Corresponding: zhongguan115@163.com (Z.-G.S.); xdg@tust.edu.cn (D.-G.X.); Tel./Fax: +86-0632-3786399 (Z.G.S.); Tel./Fax: +86-022-60600019 (D.G.X.)
† These authors contributed equally to this work.

Table S1. Oligonucleotides used in this study for the construction of gene deletions.

Name	5′–3′ DNA Sequence
	**Oligos used for construction of gene deletion cassettes**
SST1-U-F	GGTCCCGCTTTCCGATTT
SST1-U-R	CCTGCAGCGTACGAAGCTTCAGCTGGTGCGACTCATAGTGCTGTTATT
SST1-Kan-F	AATAACAGCACTATGAGTCGCACCAGCTGAAGCTTCGTACGCTGCAGG
SST1-Kan-R	AATAGATGTTGGGTCACAGCATAGGCCACTAGTGGATCTGATA
SST1-D-F	TATCAGATCCACTAGTGGCCTATGCTGTGACCCAACATCTATT
SST1-D-R	TTTCAGACACGACCAAA
SDT1-U-F	CCCTGTTCTGTTCATCGC
SDT1-U-R	CCTGCAGCGTACGAAGCTTCAGCTGGCACAATGGAGTGCGTCT
SDT1-Kan-F	AGACGCACTCCATTGTGCCAGCTGAAGCTTCGTACGCTGCAGG
SDT1-Kan-R	AGGTGCGACAATGTTCAGTGGCATAGGCCACTAGTGGATCTGATA
SDT1-D-F	TATCAGATCCACTAGTGGCCTATGCCACTGAACATTGTCGCACCT
SDT1-D-R	TGAAGCGGACATGGAAAG
SSP1-U-F	GGCAGCCTCACTAATCT
SSP1-U-R	CCTGCAGCGTACGAAGCTTCAGCTGTGTCCTTGGTCTGTTCT
SSP1-Kan-F	AGAACAGACCAAGGACACAGCTGAAGCTTCGTACGCTGCAGG
SSP1-Kan-R	CAAATACAGATACCACCCGCATAGGCCACTAGTGGATCTGATA
SSP1-D-F	TATCAGATCCACTAGTGGCCTATGCGGGTGGTATCTGTATTTG
SSP1-D-R	TTCATCTCGCTGTCTACT
SDP1-U-F	TTCTAACTCAGGAGCCAATA
SDP1-U-R	CCTGCAGCGTACGAAGCTTCAGCTGCAGCCAACTCACCCAGAG
SDP1-Kan-F	CTCTGGGTGAGTTGGCTGCAGCTGAAGCTTCGTACGCTGCAGG
SDP1-Kan-R	GCTCTACGGATTCACTGGGCATAGGCCACTAGTGGATCTGATA
SDP1-D-F	TATCAGATCCACTAGTGGCCTATGCCCAGTGAATCCGTAGAGC
SDP1-D-R	ATGACAACCCAGACAAGG
SSA9-U-F	CCCATGGGAAGTCATAG

Name	5′–3′ DNA Sequence
SSA9-U-R	CCTGCAGCGTACGAAGCTTCAGCTGTGTTTCCTACCCCAATG
SSA9-Kan-F	CATTGGGGTAGGAAACACAGCTGAAGCTTCGTACGCTGCAGG
SSA9-Kan-R	CGCGACTATAACTTTTTCCCGCATAGGCCACTAGTGGATCTGATA
SSA9-D-F	TATCAGATCCACTAGTGGCCTATGCGGGAAAAAGTTATAGTCGCG
SSA9 -D-R	CGCTACCAAAAAGAGGC
SDA9-U-F	CGACTCAATGACTGCTGGTT
SDA 9-U-R	CCTGCAGCGTACGAAGCTTCAGCTGGAAAAGGCTGGGCGATTA
SDA9-Kan-F	TAATCGCCCAGCCTTTTCCAGCTGAAGCTTCGTACGCTGCAGG
SDA9-Kan-R	TCTTATCCAACCCTCAAAGCATAGGCCACTAGTGGATCTGATA
SDA9-D-F	TATCAGATCCACTAGTGGCCTATGCTTTGAGGGTTGGATAAGA
SDA 9-D-R	GCATAGGCGATGGTGAGT
SSA10-U-F	TATGAGCCGTTCGTATGG
SSA10-U-R	CAGCGTACGAAGCTTCAGCTGAGTGAGCTGCCGCTGTAG
SSA10-Kan-F	CTACAGCGGCAGCTCACTCAGCTGAAGCTTCGTACGCTG
SSA10-Kan-R	TGTGGTAGTGGTAAAATAGCATAGGCCACTAGTGGATCTG
SSA10-D-F	CAGATCCACTAGTGGCCTATGCTATTTTACCACTACCACA
SSA10-D-R	ATACTCTTACAGCATCCT
SDA10-U-F	AGTTGACGCGACTTCTGT
SDA10-U-R	CCTGCAGCGTACGAAGCTTCAGCTGTGTAGGTTCCGATCACTA
SDA10-Kan-F	TAGTGATCGGAACCTACACAGCTGAAGCTTCGTACGCTGCAGG
SDA10-Kan-R	TGTCATCTGTGCTGCACCGCATAGGCCACTAGTGGATCTGATA
SDA10-D-F	TATCAGATCCACTAGTGGCCTATGCGGTGCAGCACAGATGACA
SDA10-D-R	CGCCCACAAGTTTCTATTTT
	**Oligos used for diagnostic purposes**
SST1-1-F	CGATGGACCCGAATCTCC
SST1-1-KanR	CTCAGTGGCAAATCCTAA
SST1-2-KanF	GGATTTGCCACTGAGGTT
SST1-2-R	GCCGACCAAGTTACCAGA
SDT1-1-F	CGCAGCATAGTGTTAGTG
SDT1-1-KanR	TTCCGTCAGCCAGTTTAG
SDT1-2-KanF	GACTAAACTGGCTGACGG
SDT1-2-R	AGGTCTCGGTTGCTCTTA
SSP1-1-F	GATCCGGTGGAATCTATG
SSP1-1-KanR	TTGATGGTCGGAAGAGGC
SSP1-2-KanF	CGTATGTGAATGCTGGTC
SSP1-2-R	TTTAGTTATCGGAGTGGC
SDP1-1-F	GGGGAACTTGATTTGGTA
SDP1-1-KanR	CGATAGATTGTCGCACCT
SDP1-2-KanF	ATGCGTCAATCGTATGTG
SDP1-2-R	TACCATCTTGGCTTTGTG
SSA9-1-F	GGCTCTCTGTAAATACCC
SSA9-1-KanR	TTGATGGTCGGAAGAGGC
SSA9-2-KanF	CGTATTTCGTCTCGCTCA
SSA9-2-R	GATACATCACGCGCTTC
SDA9-1-F	GGCTCTCTGTAAATACCC
SDA9-1-KanR	TTGATGGTCGGAAGAGGC
SDA9-2-KanF	ACCGGATTCAGTCGTCAC
SDA9-2-R	TGCCACTACCGATTCTTT
SSA10-1-F	AGGGTGTGAAAAGGTGGC
SSA10-1-KanR	TTGATGGTCGGAAGAGGC

Name	5′–3′ DNA Sequence
SSA10-2-KanF	CGTATTTCGTCTCGCTCA
SSA10-2-R	GCGAGTAACTAAACAAAT
SDA10-1-F	TTAACTGATCAACCCTCT
SDA10-1-KanR	GTGAGTCTTTTCCTTACC
SDA10-2-KanF	ATGCGTCAATCGTATGTG
SDA10-2-R	TTGGTATGGTTGGCTTAT
K-U	CAGCTGAAGCTTCGTACGC
K-D	GCATAGGCCACTAGTGGATCTG
**For real-time qPCR**	
GAT1-F	CACTTCCTGCTCGTCCTC
GAT1-R	GTATTATTGGCGATGCTG
GAP1-F	GTCCAACAGGTGGTTACAT
GAP1-R	AGCGGTGACGAAGACAGA
ARO9-F	AATGTGGAGGCTACAGGA
ARO9-R	ACCAATTATCGAGCAACT
ARO10-F	AACCCTGGTGATGTTGTC
ARO10-R	ATGTGAGCGTTTGAGTGG
ARO80-F	TGAGACCTCCACCTACCC
ARO80-R	TCAATCGCCTCCTCTTTA
UBC6-F	GGACCTGCGGATACTCCTTAC
UBC6-R	TAATCGTGTGTTGGGCTTGA

Deletion or disruption was confirmed by PCR using diagnostic primers 1-F combined with 1-KanR and 2-KanF combined with 2-R.
